# Medication adherence barriers and digital support among Saudi adults with chronic conditions

**DOI:** 10.1038/s41598-026-40815-w

**Published:** 2026-02-25

**Authors:** Mohammed M. Aldurdunji, Ohood K. Almuzaini, Abdulelah A. Alfattani, Abdulrahman I. Almaghrabi, Ahmad E. Alqurashy, Khaled T. Alharbi, Saeed H. Alzahrani, Moayed M. Kenkar, Mutep H. Aljahdali, Saad M. Wali

**Affiliations:** 1 Pharmaceutical Practices Department, College of Pharmacy, Umm Al-Qura University, Makkah, Saudi Arabia; 2 Pharmacology and Toxicology Department, College of Pharmacy, Umm Al- Qura University, Makkah, Saudi Arabia; 3 Pharmaceutical Sciences Department, College of Pharmacy, Umm Al-Qura University, Makkah, Saudi Arabia; 4 College of Pharmacy, Umm Al-Qura University, Makkah, Saudi Arabia

**Keywords:** Medication adherence, Chronic disease, Digital health, Saudi Arabia, Patient behavior, Diseases, Health care, Medical research, Risk factors

## Abstract

Medication adherence is essential for managing chronic diseases, yet non-adherence remains a significant public health issue globally and regionally. In Saudi Arabia, behavioral and systemic barriers to adherence are understudied, particularly regarding the role of digital tools. To evaluate the knowledge, attitudes, and practices related to medication adherence among adults with chronic conditions in Saudi Arabia and to identify barriers and facilitators influencing adherence, including digital support tools. A cross-sectional survey was conducted in Q1 2025 across multiple regions in Saudi Arabia. Adults with chronic illnesses were recruited using convenience sampling. A validated electronic questionnaire assessed knowledge, attitudes, intentional non-adherence, and digital tool use. Data were analyzed using descriptive statistics and multivariate logistic regression to identify predictors of non-adherence. A total of 950 participants were included (mean age: 48.6 years; 73.9% female). Although 65.9% strongly agreed on the importance of medication, 30.0% reported intentional non-adherence. Middle-aged adults (50–59 years) and males were more likely to alter or skip medications. Forgetfulness and dislike of medications were the most reported reasons. Only 24.2% used digital tools, primarily for reminders. Gender differences were observed in adherence strategies, with women more likely to use organizers and men relying on family or mobile apps. Positive beliefs about medication necessity were significantly associated with higher adherence and digital tool engagement. Medication adherence remains suboptimal among adults with chronic conditions in Saudi Arabia, influenced by demographic, behavioral, and technological factors. Tailored interventions addressing gender-specific needs, behavioral motivations, and digital literacy are warranted to improve long-term adherence and disease outcomes.

**Supplementary Information:**

The online version contains supplementary material available at 10.1038/s41598-026-40815-w.

## Introduction

Medication adherence is critical for the management of chronic conditions, including diabetes, hypertension, and cardiovascular diseases. It is defined as the degree to which a patient’s medication-taking behavior aligns with the prescribed health guidance agreed upon with healthcare providers. The significance of adherence is underscored by its direct relationship with effective disease management, symptom control, stabilization of chronic conditions, and prevention of complications^1,2^. Non-adherence remains a pervasive challenge globally, affecting nearly half of patients with chronic diseases. In high-income countries such as the United States, approximately 40% of individuals with cardiovascular conditions report behaviors such as skipping doses or delaying refills^3,4^. Similar challenges are mirrored in low- to middle-income regions, including sub-Saharan Africa and Southeast Asia, where medication access, financial constraints, and sociocultural factors contribute to poor adherence^5^.

In the Middle East and North Africa (MENA) region, particularly within the Gulf Cooperation Council (GCC) countries such as Saudi Arabia, the rising prevalence of chronic diseases necessitates a deeper examination of medication adherence behaviors. Reports from these settings indicate suboptimal adherence among patients with diabetes and cardiovascular diseases, with contributing factors such as limited health literacy, regimen complexity, and poor patient–provider communication^6^. Many individuals encounter practical and behavioral barriers, including forgetfulness, skepticism about medication necessity, and misunderstandings about treatment benefits. Despite relatively well-resourced healthcare systems in the GCC, challenges such as lifestyle transitions and high polypharmacy rates persist^7^.

The economic consequences of non-adherence further burden healthcare systems, as poor adherence is associated with increased disease progression, higher hospitalization rates, and elevated healthcare costs due to intensified treatment needs (Cutler et al., 2018). These outcomes result from a complex interplay between individual-level factors—such as forgetfulness, side effect concerns, and low perceived treatment need—and broader systemic challenges, including drug costs, fragmented access, and insufficient follow-up (Shams et al., 2020; Unni et al., 2019). The multifactorial etiology of non-adherence reinforces the importance of a comprehensive strategy.

Various interventions have been explored to address this issue, including patient-centered counseling, health education, and medication regimen simplification^8^. In recent years, digital tools such as mobile apps, electronic reminders, and SMS-based prompts have gained attention as adherence-enhancing strategies. Nonetheless, the adoption and impact of these technologies remain inconsistent due to usability limitations, lack of integration into routine clinical care, and disparities in digital literacy^7,9^. These limitations are particularly pertinent in the Saudi context, where technology access and clinical engagement with digital health tools remain variable^10^.

Given the complexity of medication adherence, particularly in the context of chronic disease management, it is essential to investigate both individual and systemic factors that influence adherence behaviors. While adherence challenges are often most pronounced among individuals with chronic illnesses, understanding the behaviors and perceptions of adults without chronic conditions also provides valuable context for public-health interventions and digital-health adoption.

Accordingly, this study aims to systematically assess the levels of knowledge, attitudes, and behaviors related to medication adherence among adults in Saudi Arabia, with a focus on those living with chronic conditions. Adults without chronic illnesses were also included to allow comparison of adherence attitudes and digital-tool use across different health backgrounds. In addition, the study seeks to identify the personal, structural, and technological barriers that may affect adherence, with particular attention to the perceived role of digital adherence tools and support systems. The findings are intended to inform the development of targeted, patient-centered strategies to enhance medication adherence and promote sustained disease control.

## Methods

### Study design

This descriptive, cross-sectional behavioral study assessed medication-taking beliefs, adherence behaviors, and the use of digital support tools among adults in Saudi Arabia, with a particular focus on those living with chronic conditions. The design aimed to explore behavioral patterns, perceptions of medication importance, and the perceived role of external support and digital technologies in promoting adherence.

### Study population and sampling

The study population consisted of adults aged 18 years and older residing in Saudi Arabia. Participants were eligible if they were currently prescribed one or more medications, whether for short-term or long-term use, or had prior experience with medication use. This inclusive approach allowed for comparison between adults with chronic illnesses and those without chronic conditions, providing insight into how health status influences medication-taking attitudes and technology adoption. Individuals unable to provide informed consent or with cognitive impairment were excluded. Convenience sampling was used to recruit participants from healthcare institutions, public venues, and online platforms across multiple regions. Participants reported the number of chronic conditions they had (none, one, two, or three or more); specific disease types were not collected to maintain survey brevity and respondent engagement.

### Data collection

Data were collected using a structured, self-administered questionnaire developed by the research team to examine medication-taking behaviors, perceived barriers, and the role of digital tools in supporting adherence. The content and structure of the questionnaire were informed by previously published studies on medication adherence and digital-health engagement in Saudi Arabia and comparable contexts^7,10–12^. The instrument included sections on demographic characteristics, beliefs about medication importance, personal adherence behaviors such as forgetfulness or intentional skipping, perceived barriers to consistent medication use, and engagement with mobile or electronic reminder tools.

The draft instrument underwent expert review by faculty members in pharmacy practice to ensure content validity and contextual relevance, followed by a pilot test with a small group of adults to assess clarity and comprehension. Minor revisions were made based on feedback to improve item wording and flow. The final version was distributed electronically via Google Forms. Data collection took place between January and March 2025, with dissemination through university, healthcare, and community networks across multiple regions of Saudi Arabia. The final English version of the questionnaire used in this study is provided as *Supplementary Material 1*.

### Sample size

The minimum required sample size was calculated using a 95% confidence level and a 5% margin of error, assuming a conservative estimated prevalence of 50% for non-adherence behaviors. A 20% buffer was added to account for non-response or incomplete entries. The final number of completed responses was 949, which exceeded the targeted sample size, thereby enhancing the statistical power and representativeness of the analysis.

### Ethics

Ethical approval for the study was obtained from the Biomedical Research Ethics Committee at Umm Al-Qura University (Approval No. HAPO-02-K-012-2025-06-2848). The study protocol was reviewed and found to be in full compliance with the principles of ethical scientific research. All methods were performed in accordance with the relevant guidelines and regulations and in accordance with the Declaration of Helsinki. The principal investigator was authorized to initiate research activities within university-affiliated facilities, regional centers, and hospitals. Participation was voluntary, and informed consent was obtained from all participants prior to inclusion. All responses were anonymized to protect participant confidentiality.

### Data analysis

All statistical analyses were performed using RStudio (version 2024.9.1.394, Boston, MA, USA) with R version 4.4.2. Descriptive statistics were used to summarize demographic characteristics, patterns of medication adherence, and the role of technology. Categorical variables were presented as frequencies and percentages. Group differences between males and females were assessed using Fisher’s exact test. To identify factors associated with intentional non-adherence and technology adoption, penalized binary logistic regression analyses were conducted. Odds ratios (ORs) with 95% confidence intervals (CIs) and corresponding p-values were reported. Statistical significance was set at a two-tailed p-value of less than 0.05.

## Results

### Demographic characteristics and awareness and knowledge of medication adherence

Of the 949 participants, the majority were female (73.9%), and the most common age group was 40 to 49 years (29.6%). Most participants resided in the Western region (59.5%) and held a bachelor’s degree (47.6%). Two-thirds (66.5%) were currently receiving prescribed medications, and the vast majority strongly agreed (65.9%) that medications are important for maintaining health. Regarding the number of medications received daily, 42.6% reported taking one to two medications.

Approximately one-quarter of respondents (25.0%) reported having no chronic condition, while nearly half (47.9%) reported one and 27.1% reported two or more chronic conditions. Including both chronic and non-chronic medication users was intentional to enable comparison of adherence behaviors and digital tool engagement across different health backgrounds. This broader inclusion provides insight into general medication-taking patterns in the Saudi population while also allowing subgroup analysis by chronic condition status. Notably, participants with two chronic conditions were significantly less likely to adopt technology compared with those without chronic conditions (OR = 0.22, 95% CI 0.06–0.59, *p* = 0.002). Most participants (51.7%) stated that they themselves primarily influence decisions about their medication adherence (Table 1).

**Table 1:** Sociodemographic characteristics and awareness of medication importance among participants.

Characteristic	Description, *N* = 949
Gender	
Male	248 (26.1%)
Female	701 (73.9%)
Age (year)	
18–29	175 (18.4%)
30–39	257 (27.1%)
40–49	281 (29.6%)
50–59	129 (13.6%)
60 or more	107 (11.3%)
Region	
Eastern region	1 (0.1%)
Western region	565 (59.5%)
Northern region	5 (0.5%)
Southern region	284 (29.9%)
Central region	94 (9.9%)
Educational level	
Primary	22 (2.3%)
Middle school	46 (4.8%)
Secondary school	196 (20.7%)
Bachelor	452 (47.6%)
Post-graduate	233 (24.6%)
Currently receiving prescribed medications	631 (66.5%)
Medications are important to keep healthy	
Disagree	1 (0.2%)
Neutral	60 (9.5%)
Agree	154 (24.4%)
Strongly agree	416 (65.9%)
Number of medications received daily	
None	81 (12.8%)
1–2	269 (42.6%)
3–5	166 (26.3%)
> 5	115 (18.2%)
Number of chronic conditions	
None	158 (25.0%)
One	302 (47.9%)
Two	52 (8.2%)
Three	119 (18.9%)
The person who primarily influences the decisions about medication adherence	
None	19 (3.0%)
Myself	326 (51.7%)
Healthcare provider	175 (27.7%)
Family or caregiver	111 (17.6%)

n (%).

Values are presented as frequencies (n) and percentages (%).

### Role of technology in adherence and implementing changes in medication schedules

**Fig. 1:**
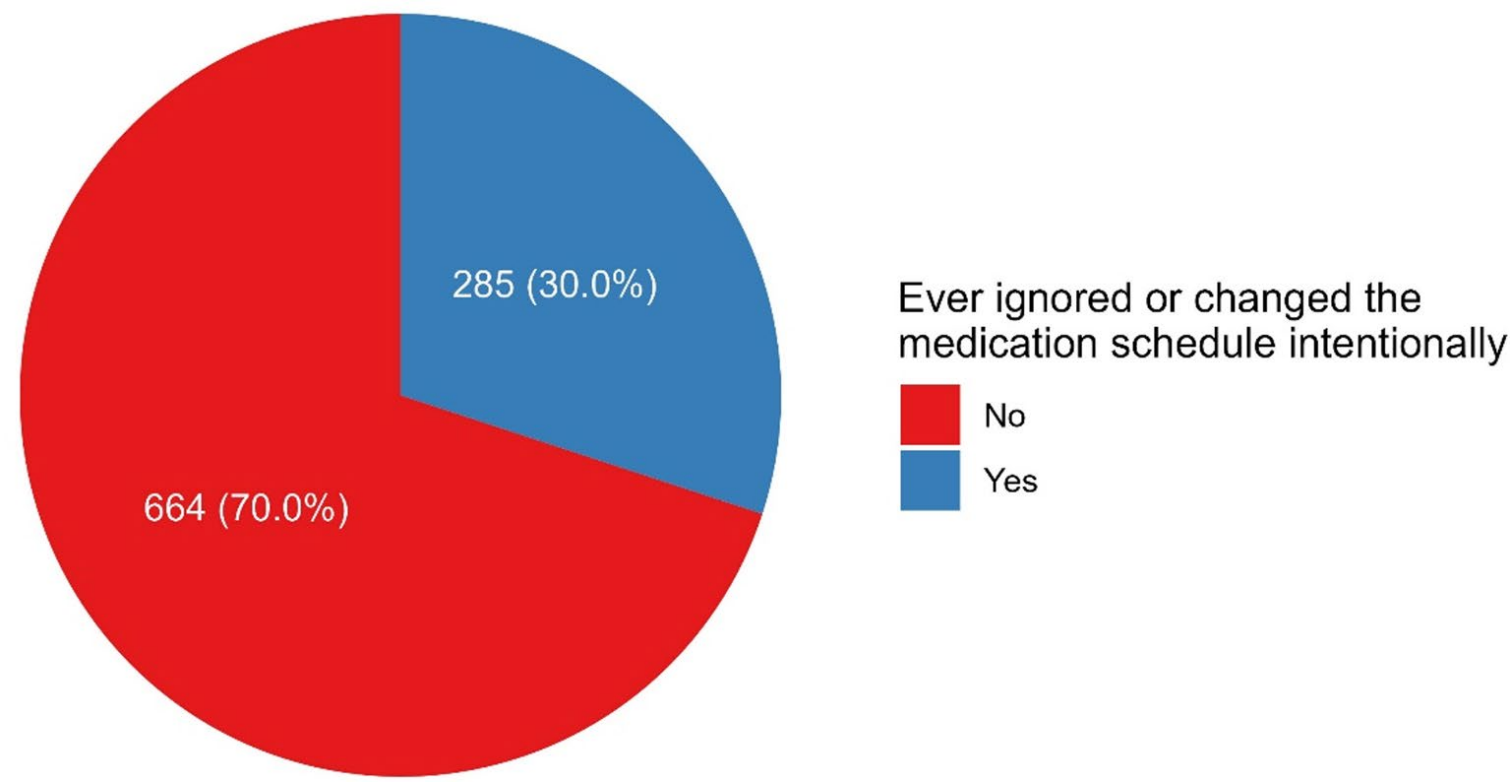
Proportion of participants reporting intentional changes to medication schedules.

Overall, 24.2% of participants reported using an app or electronic device to manage their medications. The most commonly reported useful feature was reminders and notifications (85.1%), followed by dose tracking (38.9%) and integration with healthcare providers (37.5%). Common challenges included technical issues (58.1%) and ads (49.3%, Table 2). About 30.0% reported intentionally ignoring or changing their medication schedule (Fig. 1).

**Table 2:** Use of digital tools and intentional changes to medication schedules.

Characteristic	Overall *N* = 949	Male *N* = 248	Female *N* = 701	*p*-value
Ever used an app or an electronic device to manage medications	230 (24.2%)	54 (21.8%)	176 (25.1%)	0.302
Features of the app which are most useful*				
Alerts for drug interactions	297 (31.3%)	108 (43.5%)	189 (27.0%)	< 0.001
Dose tracking	369 (38.9%)	120 (48.4%)	249 (35.5%)	< 0.001
Integration with healthcare providers	356 (37.5%)	99 (39.9%)	257 (36.7%)	0.361
Reminders and notifications	808 (85.1%)	225 (90.7%)	583 (83.2%)	0.004
Challenges faced while using the app*				
Ads	468 (49.3%)	116 (46.8%)	352 (50.2%)	0.375
Language issues	143 (15.1%)	72 (29.0%)	71 (10.1%)	< 0.001
Inadequate reminders and notifications	260 (27.4%)	60 (24.2%)	200 (28.5%)	0.214
Technical issues	551 (58.1%)	155 (62.5%)	396 (56.5%)	0.100
Ever ignored or changed the medication schedule intentionally	285 (30.0%)	92 (37.1%)	193 (27.5%)	0.006

n (%).

Fisher’s Exact Test for Count Data.

*An asterisk indicates a multiple-responce item.

Values are presented as frequencies (n) and percentages (%).

In comparisons by gender, males were more likely than females to prefer alerts for drug interactions (43.5% vs. 27.0%, *p* < 0.001), dose tracking (48.4% vs. 35.5%, *p* < 0.001), and reminders and notifications (90.7% vs. 83.2%, *p* = 0.004). Language issues were more frequently reported among males (29.0% vs. 10.1%, *p* < 0.001). Males were also more likely to report intentional changes to medication schedules (37.1% vs. 27.5%, *p* = 0.006, Table 2).

### Barriers and patterns of adherence among participants who did ignore or change the medication schedule intentionally

Among those who had intentionally altered their medication schedule (*N* = 285), the most frequently cited reason was disliking medications (57.9%), followed by forgetting to take them (49.1%). The most commonly used strategy to overcome non-adherence was using alarms or apps (69.8%), and the most common method to remember to take medications was linking it to daily activities (70.9%, Table 3).

**Table 3:** Barriers and strategies among participants reporting intentional non-adherence (*N* = 285).

Characteristic	Overall *N* = 285	Male *N* = 92	Female *N* = 193	*p*-value
The reason for skipping or altering your medication schedule*				
Due to side effects	46 (16.1%)	22 (23.9%)	24 (12.4%)	0.016
Felt better and thought it wasn’t necessary	54 (18.9%)	16 (17.4%)	38 (19.7%)	0.747
Cost of medications	16 (5.6%)	11 (12.0%)	5 (2.6%)	0.004
Forgot to take it	140 (49.1%)	22 (23.9%)	118 (61.1%)	< 0.001
Hate taking medications	165 (57.9%)	53 (57.6%)	112 (58.0%)	> 0.999
Strategies used to overcome this*				
Consulting healthcare providers	58 (20.4%)	37 (40.2%)	21 (10.9%)	< 0.001
Setting routines	78 (27.4%)	22 (23.9%)	56 (29.0%)	0.397
Using alarms or apps	199 (69.8%)	39 (42.4%)	160 (82.9%)	< 0.001
Seeking support from family or caregivers	7 (2.5%)	4 (4.3%)	3 (1.6%)	0.218
How do you remember to take your medications on time? *				
I do not have a specific strategy	24 (8.4%)	11 (12.0%)	13 (6.7%)	0.171
I keep my medications in a visible place	62 (21.8%)	9 (9.8%)	53 (27.5%)	< 0.001
I rely on memory	43 (15.1%)	7 (7.6%)	36 (18.7%)	0.014
I link it to daily activities	202 (70.9%)	69 (75.0%)	133 (68.9%)	0.330
I rely on family or caregivers	9 (3.2%)	7 (7.6%)	2 (1.0%)	0.006
I set an alarm or use a reminder app	60 (21.1%)	12 (13.0%)	48 (24.9%)	0.029
Why do you prefer reminders from family or caregivers?				> 0.999
I am not comfortable with technology	1 (11.1%)	1 (14.3%)	0 (0.0%)	
I trust them more than technology	4 (44.4%)	3 (42.9%)	1 (50.0%)	
It feels more personal	4 (44.4%)	3 (42.9%)	1 (50.0%)	
It helps me stay connected with my caregivers	0 (0.0%)	0 (0.0%)	0 (0.0%)	
How do you feel about being reminded by others?				> 0.999
1 - Very uncomfortable	4 (44.4%)	3 (42.9%)	1 (50.0%)	
2 - Somewhat uncomfortable	0 (0.0%)	0 (0.0%)	0 (0.0%)	
3 - Neutral	1 (11.1%)	1 (14.3%)	0 (0.0%)	
4 - Somewhat comfortable	4 (44.4%)	3 (42.9%)	1 (50.0%)	
5 - Very comfortable	0 (0.0%)	0 (0.0%)	0 (0.0%)	
What would make you consider using an alternative method, like an app? *				
Assurance of privacy and security	3 (33.3%)	1 (14.3%)	2 (100.0%)	0.083
Improved understanding of apps	8 (88.9%)	6 (85.7%)	2 (100.0%)	> 0.999
Support from healthcare providers	1 (11.1%)	0 (0.0%)	1 (50.0%)	0.222
Have you missed a dose due to disruptions in your routine?				> 0.999
No	88 (59.5%)	14 (60.9%)	74 (59.2%)	
Yes, occasionally	57 (38.5%)	9 (39.1%)	48 (38.4%)	
Yes, frequently	3 (2.0%)	0 (0.0%)	3 (2.4%)	
What would make you consider using an alternative method, like an app?				> 0.999
Ease of use	145 (98.0%)	23 (100.0%)	122 (97.6%)	
Assurance of privacy and security	3 (2.0%)	0 (0.0%)	3 (2.4%)	

n (%).

Fisher’s Exact Test for Count Data.

*An asterisk indicates a multiple-response item.

Values are presented as frequencies (n) and percentages (%).

Gender differences revealed that males were more likely to skip medications due to side effects (23.9% vs. 12.4%, *p* = 0.016) and cost (12.0% vs. 2.6%, *p* = 0.004), while females were more likely to forget taking medications (61.1% vs. 23.9%, *p* < 0.001). Males were also more likely to consult healthcare providers (40.2% vs. 10.9%, *p* < 0.001), whereas females more often used alarms or apps (82.9% vs. 42.4%, *p* < 0.001). Females were more likely to keep medications visible (27.5% vs. 9.8%, *p* < 0.001), rely on memory (18.7% vs. 7.6%, *p* = 0.014), or set reminders (24.9% vs. 13.0%, *p* = 0.029). Males more often relied on family or caregivers (7.6% vs. 1.0%, *p* = 0.006, Table 3).

### Personal adherence practices and barriers to future medication use among participants who did not ignore or change the medication schedule intentionally

Among those who had not altered their medication schedule (*N* = 664), the most common reasons for skipping medications were forgetting to take them (75.2%) and feeling better (53.0%). Common strategies included using alarms or apps (63.9%), consulting healthcare providers (47.7%), and setting routines (43.5%). Almost half (44.3%) used reminders or alarms, and 55.7% would manage multiple medications using alarms (Table 4).

**Table 4:** Adherence practices and perceived barriers among participants without intentional non-adherence (*N* = 664).

Characteristic	Overall *N* = 664	Male *N* = 156	Female *N* = 508	*p*-value
The reason for skipping or altering your medication schedule*				
Cost of medications	86 (13.0%)	12 (7.7%)	74 (14.6%)	0.029
Felt better and thought it wasn’t necessary	352 (53.0%)	79 (50.6%)	273 (53.7%)	0.522
Forgot to take	499 (75.2%)	114 (73.1%)	385 (75.8%)	0.525
Hate taking medications	146 (22.0%)	27 (17.3%)	119 (23.4%)	0.122
Side effects	105 (15.8%)	39 (25.0%)	66 (13.0%)	< 0.001
Strategies used to overcome this*				
Consulting healthcare providers	317 (47.7%)	56 (35.9%)	261 (51.4%)	< 0.001
Seeking support from family or caregivers	207 (31.2%)	51 (32.7%)	156 (30.7%)	0.693
Setting routines	289 (43.5%)	49 (31.4%)	240 (47.2%)	< 0.001
Use alarms or apps	424 (63.9%)	117 (75.0%)	307 (60.4%)	< 0.001
How do you remember to take your medications on time?				< 0.001
I don’t have a specific strategy	48 (7.2%)	9 (5.8%)	39 (7.7%)	
I keep my medications in a visible place	115 (17.3%)	26 (16.7%)	89 (17.5%)	
I link it to daily activities	110 (16.6%)	11 (7.1%)	99 (19.5%)	
I rely on family or caregivers	16 (2.4%)	11 (7.1%)	5 (1.0%)	
I rely on memory	81 (12.2%)	34 (21.8%)	47 (9.3%)	
I set an alarm or use a reminder app	294 (44.3%)	65 (41.7%)	229 (45.1%)	
If you were prescribed multiple medications, how would you approach managing them? *				
Asking family or caregivers for help	57 (8.6%)	26 (16.7%)	31 (6.1%)	< 0.001
Linking them to daily routines	368 (55.4%)	95 (60.9%)	273 (53.7%)	0.119
Using visual cues	30 (4.5%)	11 (7.1%)	19 (3.7%)	0.119
I rely on memory	162 (24.4%)	59 (37.8%)	103 (20.3%)	< 0.001
Setting alarms or reminders	370 (55.7%)	80 (51.3%)	290 (57.1%)	0.231
Using a pill organizer	232 (34.9%)	31 (19.9%)	201 (39.6%)	< 0.001
If a healthcare provider recommended a specific app to manage medications, would you try it?				0.001
No	35 (5.3%)	2 (1.3%)	33 (6.5%)	
Yes	583 (87.8%)	137 (87.8%)	446 (87.8%)	
Unsure	46 (6.9%)	17 (10.9%)	29 (5.7%)	
If a medication caused side effects, how would you handle the situation?				0.124
Continue taking it until my next doctor’s visit	58 (8.7%)	19 (12.2%)	39 (7.7%)	
Look for alternative remedies or supplements	2 (0.3%)	1 (0.6%)	1 (0.2%)	
Stop taking the medication and inform my doctor	604 (91.0%)	136 (87.2%)	468 (92.1%)	
What would be your biggest concern if you had to take medications regularly?				< 0.001
Cost of medications	22 (3.3%)	4 (2.6%)	18 (3.5%)	
Fear of dependency or addiction	12 (1.8%)	10 (6.4%)	2 (0.4%)	
Forgetting to take the medications	342 (51.5%)	100 (64.1%)	242 (47.6%)	
Immunosuppression	204 (30.7%)	22 (14.1%)	182 (35.8%)	
Lack of trust in medications	84 (12.7%)	20 (12.8%)	64 (12.6%)	
If you needed support for medication adherence, whom would you turn to? *				
Apps or reminders	184 (27.7%)	80 (51.3%)	104 (20.5%)	< 0.001
I would manage it on my own	310 (46.7%)	66 (42.3%)	244 (48.0%)	0.233
Family or friends	167 (25.2%)	62 (39.7%)	105 (20.7%)	< 0.001
Healthcare provider	182 (27.4%)	37 (23.7%)	145 (28.5%)	0.260
Do you believe that educating people about medication adherence is important?				0.027
Neutral	37 (5.6%)	6 (3.8%)	31 (6.1%)	
Agree	78 (11.7%)	10 (6.4%)	68 (13.4%)	
Strongly agree	549 (82.7%)	140 (89.7%)	409 (80.5%)	
What type of information about medications would you find most useful? *				
How medications work in the body	370 (55.7%)	104 (66.7%)	266 (52.4%)	0.002
Importance of adherence to prescribed schedules	409 (61.6%)	105 (67.3%)	304 (59.8%)	0.110
Tools and strategies for managing medications	119 (17.9%)	21 (13.5%)	98 (19.3%)	0.120
Side effects and how to manage them	369 (55.6%)	82 (52.6%)	287 (56.5%)	0.408

n (%).

Fisher’s Exact Test for Count Data.

*An asterisk indicates a multiple-choice item.

Values are presented as frequencies (n) and percentages (%).

### Gender comparisons revealed that females were more likely to report side effects as a barrier

(13.0% vs. 25.0%, *p* < 0.001), and more frequently used routines (47.2% vs. 31.4%, *p* < 0.001) or consulted healthcare providers (51.4% vs. 35.9%, *p* < 0.001). Females were also more likely to use pill organizers (39.6% vs. 19.9%, *p* < 0.001) and less likely to rely on memory (20.3% vs. 37.8%, *p* < 0.001). When seeking support, males more frequently preferred apps (51.3% vs. 20.5%, *p* < 0.001) and family or friends (39.7% vs. 20.7%, *p* < 0.001). Females were more likely to strongly agree on the importance of medication education (80.5% vs. 89.7%, *p* = 0.027) and preferred learning how medications work (52.4% vs. 66.7%, *p* = 0.002, Table 4).

### Association between intentional non-adherence to medications and strategies used to remember taking medications on time

Participants who reported intentionally ignoring or changing their medication schedule were more likely to link medication intake to daily activities (70.9% vs. 16.6%, *p* < 0.001), while those who did not engage in intentional non-adherence were more likely to set alarms or use reminder apps (44.3% vs. 21.1%, *p* < 0.001). No statistically significant differences were observed between the groups in the use of visible placement, reliance on memory, caregiver support, or having no specific strategy (Table 5).

**Table 5:** Association between intentional non-adherence and reminder strategies.

Characteristic	Ever ignored or changed the medication schedule intentionally		*p*-value
	No *N* = 664	Yes *N* = 285	
Strategies used to remember taking medications on time			
I do not have a specific strategy	48 (7.2%)	24 (8.4%)	0.525
I keep my medications in a visible place	115 (17.3%)	62 (21.8%)	0.108
I rely on memory	81 (12.2%)	43 (15.1%)	0.226
I link it to daily activities	110 (16.6%)	202 (70.9%)	< 0.001
I rely on family or caregivers	16 (2.4%)	9 (3.2%)	0.509
I set an alarm or use a reminder app	294 (44.3%)	60 (21.1%)	< 0.001

### Regression analysis for predicting intentional non-adherence to medications

Females were significantly less likely than males to intentionally change or ignore their medication schedule (OR = 0.51, 95% CI, 0.35 to 0.75, *p* < 0.001). Compared to participants aged 18 to 29, those aged 30 to 39 (OR = 3.37, 95% CI, 2.02 to 5.74, *p* < 0.001) and those aged 50 to 59 (OR = 8.31, 95% CI, 4.70 to 15.0, *p* < 0.001) had significantly higher odds of non-adherence. Additionally, participants who were currently receiving prescribed medications had increased odds of intentional non-adherence (OR = 1.72, 95% CI, 1.21 to 2.46, *p* = 0.003). Other sociodemographic variables were not significantly associated with non-adherence (Table 6).

**Table 6:** Predictors of intentional non-adherence: logistic regression results.

Characteristic	OR	95% CI	*p*-value
Gender			
Male	Reference	Reference	
Female	0.51	0.35, 0.75	< 0.001
Age (year)			
18 to 29	Reference	Reference	
30 to 39	3.37	2.02, 5.74	< 0.001
40 to 49	1.16	0.68, 1.99	0.590
50 to 59	8.31	4.70, 15.0	< 0.001
60 or more	1.16	0.55, 2.41	0.690
Region			
Eastern region	Reference	Reference	
Western region	1.54	0.08, 229	0.788
Northern region	0.08	0.00, 20.3	0.300
Southern region	0.92	0.05, 137	0.961
Central region	1.57	0.08, 237	0.779
Educational level			
Primary	Reference	Reference	
Middle school	0.37	0.07, 2.06	0.248
Secondary school	2.57	0.83, 10.4	0.105
Bachelor	1.62	0.54, 6.45	0.415
Post-graduate	0.67	0.21, 2.73	0.540
Currently receiving prescribed medications			
No	Reference	Reference	
Yes	1.72	1.21, 2.46	0.003
Ever used an app or an electronic device to manage medications			
No	Reference	Reference	
Yes	0.78	0.52, 1.14	0.202

OR = Odds Ratio; CI = Confidence Interval. Results are based on penalized binary logistic regression analysis.

Statistical significance was set at *p* < 0.05 (two-tailed).

### Regression analysis for predicting technology adoption

Participants aged 30 to 39 (OR = 0.47, 95% CI, 0.25 to 0.90, *p* = 0.022) and 40 to 49 (OR = 0.40, 95% CI, 0.22 to 0.74, *p* = 0.004) were significantly less likely to adopt technology compared to those aged 18 to 29. Participants with a middle school education were more likely to adopt technology than those with primary education (OR = 12.6, 95% CI, 3.12 to 63.4, *p* < 0.001). Those who expressed agreement (OR = 0.04, 95% CI, 0.00 to 0.85, *p* = 0.040) or strong agreement (OR = 0.02, 95% CI, 0.00 to 0.47, *p* = 0.017) with the belief that medications are important for health were significantly more likely to adopt technology than those who disagreed. Participants with two chronic conditions were significantly less likely to adopt technology compared to those without chronic conditions (OR = 0.22, 95% CI, 0.06 to 0.59, *p* = 0.002, Table 7).

**Table 7:** Predictors of digital tool adoption for medication management.

Characteristic	OR	95% CI	*p*-value
Gender			
Male	Reference	Reference	
Female	1.08	0.67, 1.75	0.754
Age (year)			
18 to 29	Reference	Reference	
30 to 39	0.47	0.25, 0.90	0.022
40 to 49	0.40	0.22, 0.74	0.004
50 to 59	0.55	0.26, 1.14	0.108
60 or more	0.68	0.31, 1.45	0.324
Region			
Western region	Reference	Reference	
Northern region	0.31	0.00, 3.28	0.381
Southern region	1.11	0.72, 1.70	0.642
Central region	0.63	0.29, 1.28	0.207
Educational level			
Primary	Reference	Reference	
Middle school	12.6	3.12, 63.4	< 0.001
Secondary school	0.64	0.19, 2.71	0.510
Bachelor	1.33	0.42, 5.45	0.642
Post-graduate	0.88	0.26, 3.73	0.847
Currently receiving prescribed medications			
No	Reference	Reference	
Yes	1.05	0.62, 1.83	0.865
Medications are important to keep healthy			
Disagree	Reference	Reference	
Neutral	0.03	0.00, 0.79	0.036
Agree	0.04	0.00, 0.85	0.040
Strongly agree	0.02	0.00, 0.47	0.017
Number of chronic conditions			
None	Reference	Reference	
One	0.81	0.49, 1.37	0.434
Two	0.22	0.06, 0.59	0.002
Three	0.55	0.28, 1.05	0.071

OR = Odds Ratio; CI = Confidence Interval. Results are based on penalized binary logistic regression analysis.

Statistical significance was set at *p* < 0.05 (two-tailed).

## Discussion

Medication adherence remains a persistent challenge in chronic disease management, influenced by behavioral, demographic, and systemic factors. In this study, 285 participants (30%) reported intentionally skipping or altering their prescribed medication schedule, despite 65.9% expressing strong agreement that medications were important for maintaining health. This discrepancy reflects a notable knowledge–behavior gap, consistent with World Health Organization estimates that nearly half of patients in developed countries do not adhere to long-term therapies^11^. Similarly, Movahedinejad and Adib-Hajbaghery reported that strong beliefs in medication necessity often coexist with inconsistent adherence, commonly due to side effects and forgetfulness^13^.

The findings align with global evidence indicating that medication adherence remains suboptimal among patients with chronic diseases. Reviews from Europe, North America, and Asia report comparable non-adherence rates, with behavioral factors such as forgetfulness, perceived necessity, and regimen complexity emerging as consistent determinants^14–16^. Systematic reviews have further demonstrated that adherence is influenced by psychological, socioeconomic, and technological factors^17–19^. Psychological distress, including depression and anxiety, is associated with lower adherence^20,21^, while socioeconomic constraints—such as healthcare access and education—also shape medication-taking behavior^22,23^. The relatively limited use of digital adherence tools observed in this study compared with high-income settings likely reflects regional variation in digital literacy, language accessibility, and integration of mHealth platforms into clinical care. Additional factors such as usability challenges, limited trust in digital health technologies, and concerns about data privacy may further constrain adoption in this context. Collectively, these results emphasize the interplay between universal behavioral drivers and context-specific structural barriers to adherence.

The first major domain concerns behavioral and sociodemographic predictors of intentional non-adherence. Age and gender emerged as key determinants. Middle-aged participants, particularly those aged 50–59 years, demonstrated a markedly higher likelihood of intentional non-adherence (OR = 8.31; 95% CI, 4.70–15.0), consistent with Gast and Mathes, who linked midlife occupational and familial demands to reduced treatment regularity^15^. This pattern aligns with the Health Belief Model, which posits that perceived susceptibility and severity influence motivation to adhere, factors that may be deprioritized when competing daily responsibilities dominate^24^. Gender differences were also evident: women were significantly more adherent than men (OR = 0.51; 95% CI, 0.35–0.75), corroborating findings by Vervloet et al. and Al-Hassany et al., who attributed women’s higher adherence to stronger perceptions of medication necessity and proactive self-care^12,25^. According to the Necessity–Concerns Framework, these attitudes indicate higher necessity beliefs and fewer concerns among women, while men may require external reinforcement or structured environments to sustain adherence^26,27^.

Behavioral motivations for non-adherence also revealed gendered patterns. Among those who altered their regimen, the most common reasons were disliking medications (57.9%) and forgetting doses (49.1%), consistent with prior literature identifying these as leading barriers^6,16^. Men were more likely to cite cost and side effects, as observed by Khan et al.^28^. In contrast, women’s adherence behaviors often reflect sociocultural and psychological influences. Studies from Saudi Arabia and neighboring Gulf countries show that family obligations, cultural norms, and health-related stigma can shape women’s medication behaviors and limit their autonomy in health decisions^11,19,29^. These patterns align with the Necessity–Concerns Framework, suggesting that gender differences in adherence reflect differing perceptions of medication benefit, risk, and necessity^30,31^.

The second domain addresses technology use and intervention design. Only 24.2% of participants reported using digital tools for adherence, most commonly for reminders and dose tracking. This limited adoption parallels findings by Dou et al., who noted that patients often underuse available technologies despite acknowledging their benefits^32^. Fallatah et al. similarly identified digital literacy, accessibility, and confidence in technology as major barriers^33^. The language and usability issues observed here echo Al-Hassany et al.’s call for culturally tailored mHealth solutions^12^. Despite national initiatives such as Sehha and Wasfaty under Saudi Vision 2030, persistent challenges, including gender disparities, limited Arabic-language content, and uneven digital competence, continue to restrict engagement^34–38^. Effective adoption requires alignment between user capability, cultural relevance, and system-level support.

The limited use of digital tools observed may also reflect gendered and behavioral preferences. Women’s greater reliance on simple reminders such as alarms and organizers suggests a preference for low-complexity, habit-based solutions, while men’s dependence on family or provider support indicates lower comfort with digital autonomy. Similar trends have been observed in other Gulf populations, where men often rely on interpersonal or technology-mediated assistance^39^. These findings emphasize that digital engagement is shaped by gender roles, perceived usability, and social context. Future research using qualitative or mixed methods could further clarify the sociocultural and experiential factors underlying mHealth adoption.

Among the 664 participants who did not intentionally alter their regimen, common behavioral strategies included setting alarms and consulting healthcare providers. Women were more likely to use pill organizers and structured daily routines, consistent with Yadav et al., who demonstrated the effectiveness of organizational strategies in enhancing adherence^40^. These behaviors likely reflect women’s greater self-regulatory discipline and preference for tangible tools that support habit formation^41,42^. Men, by contrast, reported higher reliance on mobile applications and family reminders, reflecting alternative mechanisms of behavioral reinforcement^37^. Such patterns highlight the importance of tailoring interventions to different motivational and practical preferences across gender groups.

Beliefs about medication necessity further shaped both adherence and engagement with technology. Participants who viewed their medications as essential were significantly more likely to use digital tools (OR = 0.02; 95% CI, 0.00–0.47), aligning with the Necessity–Concerns Framework^43^. This relationship reflects the motivational component of the COM-B model, where positive attitudes toward treatment facilitate supportive behaviors. Nearly two-thirds of participants expressed a desire to understand how their medications work, with women more likely than men to seek this knowledge (*p* = 0.027). These findings correspond with evidence showing that greater medication literacy enhances adherence and confidence in self-management^7,14,43^. Educational interventions that improve pharmacological understanding may therefore complement digital adherence strategies by reinforcing motivation and perceived control.

The predominance of female participants (74%) should be interpreted within the context of survey-based recruitment in Saudi Arabia, where women are more likely to engage in online health research^44–46^. While this gender imbalance may limit generalizability, it also provides valuable insight into women’s health behaviors, an area underrepresented in regional adherence studies and digital health research^47–49^. Future research employing stratified or quota-based sampling could yield more balanced data and enable comparative analysis across gender and sociodemographic groups.

This study has several limitations that should be considered when interpreting the findings. The cross-sectional design restricts causal inference between behavioral factors, digital engagement, and medication adherence. Because adherence was assessed through self-report, recall and social desirability biases may have influenced the results. The use of convenience sampling and the predominance of female participants (73.9%) may also limit generalizability, as voluntary participation can introduce self-selection bias. Nevertheless, the large sample size, regional diversity, and inclusion of both chronic and non-chronic medication users enhance the internal validity and breadth of behavioral insight. The inclusion of participants without chronic conditions was intentional to provide comparative perspectives, though this broader inclusion may have diluted condition-specific effects. Moreover, while the study captured the number of chronic conditions, it did not differentiate between specific disease types. Future research using stratified or disease-specific cohorts, probability-based sampling, and objective adherence measures such as pharmacy refill data, electronic monitoring, or app-based usage logs could further clarify causal pathways and condition-specific variations in medication adherence and digital tool adoption.

## Conclusion

This study demonstrates that medication adherence among Saudi adults with chronic conditions is shaped by an interaction of behavioral, demographic, psychological, and systemic factors. Intentional non-adherence was more common among middle-aged and male participants, while adherence-supportive behaviors such as routine formation, organizational tools, and digital reminders were more frequent among women. Although Saudi Arabia has made significant progress in developing digital health infrastructure, the adoption of mHealth tools remains limited and is influenced by literacy, usability, and sociocultural factors. The findings highlight the need for patient-centered interventions that combine behavioral education, digital innovation, and culturally appropriate support to promote sustainable medication adherence. Integrating these approaches into routine care and national eHealth strategies may contribute to achieving the goals of Saudi Vision 2030 by improving chronic disease management and public health outcomes.

## Supplementary Information

Below is the link to the electronic supplementary material.

Supplementary Material 1

## Data Availability

The datasets used and analyzed during the current study are available from the corresponding author on reasonable request.
