# Supplementary material for: Medication adherence barriers and digital support among Saudi adults with chronic conditions
Source: Sci Rep. 2026 Feb 25;16:8719. doi: 10.1038/s41598-026-40815-w (PMC12979819; doi:10.1038/s41598-026-40815-w)
Supplement: Supplementary file 1 — Supplementary Material 1 [file 41598_2026_40815_MOESM1_ESM.pdf]

Section 1: General Information

1. **Gender:** Male / Female
2. **Age:** Under 18 / 18–29 / 30–39 / 40–49 / 50–59 / 60 or older
3. **Region:** Central / Eastern / Northern / Southern / Western / Other
4. **Educational Level:** Primary / Intermediate / Secondary / Bachelor's / Postgraduate (Master's or Doctorate) / Other
5. **Occupation:** Healthcare worker / Non-healthcare worker / Retired / Unemployed / Other
6. **Do you currently take prescribed medications?** Yes / No → *If No, skip to Question 10*

Section 2: Awareness and Knowledge of Medication Adherence

7. **Do you believe that medications are necessary to maintain good health?** Strongly agree / Agree / Neutral / Disagree / Strongly disagree
8. **How many medications do you take daily?** 0 / 1–2 / 3–5 / >5
9. **How many chronic conditions are you currently managing?** 0 / 1 / 2 / 3 / 4 / >4
10. **Who primarily influences your decisions about medication adherence?** Healthcare provider / Family or caregiver / Myself / Other (specify)
11. **Have you ever deliberately skipped or altered your medication schedule?** Yes / No → *If No, skip to Question 29*

Section 3: Personal Adherence Practices

12. **What was the reason for skipping or altering your medication schedule?** (Select all that apply)
    Side effects / Felt better before completing treatment / Forgetfulness / Cost of medications / Dislike of taking medications
13. **What strategies have you used to overcome this?** (Select all that apply)
    Using apps or reminders / Establishing routines / Support from family or caregivers / Consulting healthcare providers / Other
14. **How do you usually remember to take your medications?** (Select all that apply)
    I use an app or alarm / I rely on family or caregivers / I link medication to daily routines / I keep medications in a visible place / I rely on memory / I do not follow a specific strategy

---

Branch A – App or Reminder Use

15. **Why do you choose to use an app or reminder?** (Select all that apply) Convenience / Tracking doses / Consistent reminders / Recommended by provider / Other

16. **What challenges do you face using such tools?** (Select all that apply) Technical issues / Forgetting to set reminders / Too many notifications / Language difficulties / App not meeting needs
17. **Would you recommend a medication reminder app to others? Why or why not?** (Open-ended)

Branch B – Family or Caregiver Support

18. **Why do you prefer reminders from family or caregivers?** (Select all that apply) I trust them more than technology / It feels more personal / I am uncomfortable with technology / It helps me stay connected / Other
19. **How do you feel when others remind you to take your medications?** (Likert scale: Very satisfied – Satisfied – Neutral – Dissatisfied – Very dissatisfied)
20. **What might encourage you to try another method (e.g., an app)?** (Select all that apply) Ease of use / Privacy and security / Recommendation from provider / Support for special needs

---

Branch C – Routine-Based Adherence

21. **Why do you link medication use to daily routines?** (Select all that apply) Easy to remember / Fits naturally into the day / Prefer not to use alarms / Other
22. **Have you ever missed a dose due to disruptions in your routine?** Frequently / Sometimes / Never
23. **Would you consider using a backup method (e.g., an app)?** (Select all that apply) Ease of use / Privacy and security / Provider support / Special needs

---

Branch D – Visual Cues

24. **Why do you use visual cues (e.g., placing medications where you can see them)?** (Select all that apply) Simple and effective / Doesn’t require technology / Always visible / Other
25. **Have you forgotten to take a dose despite having visual cues?** Often / Sometimes / Never
26. **Would you consider combining visual cues with another method (e.g., app or reminders)?** Yes / No

---

Branch E – Memory-Based Adherence

27. **Why do you rely solely on memory?** (Select all that apply) I find it sufficient / I prefer not to use external aids / I trust my memory / Other
28. **How often do you forget to take your medications?** Rarely / Occasionally / Frequently
29. **Would you consider using support tools such as apps or reminders?** Yes / No

---

Section 4: Barriers to Medication Adherence

30. **What challenges do you face in adhering to medications?** (Select all that apply) Forgetfulness / Cost / Side effects / Feeling better before completing treatment / Dislike of medications
31. **How do you overcome these challenges?** (Select all that apply) Using reminders or apps / Setting routines / Support from family or caregivers / Consulting healthcare providers

---

Section 5: Role of Technology in Adherence

32. **Have you ever used a mobile app or electronic device to manage medications?** Yes / No → *If No, skip to Question 34*
33. **What features of these apps are most useful to you?** (Select all that apply) Medication reminders / Dose tracking / Integration with healthcare providers / Drug interaction alerts
34. **What challenges have you faced using these apps?** (Select all that apply) Technical problems / Language issues / Too many alerts / Not user-friendly
35. **Would you consider using a medication management app in the future?** Yes / No / Not sure

---

Section 6: Hypothetical Scenarios

36. **If you were prescribed multiple medications, how would you manage them?** (Select all that apply) Using a pill organizer / Setting electronic reminders / Asking for help from family or caregivers / Linking to daily activities / Using visual cues / Relying on memory
37. **If a doctor recommended a medication app, would you try it?** Yes / No / Not sure
38. **If a medication caused side effects, how would you handle it?** Stop and inform my doctor / Continue until next visit / Adjust dose myself / Look for alternatives

---

Section 7: Future Concerns and Support

39. **If you had to take medications regularly, what would be your main concern?** Forgetting doses / Cost / Effectiveness / Fear of dependency / Weak immunity / Other
40. **Who would you turn to for help with medication adherence?** Family or friends / Healthcare provider / Apps or reminders / I would manage on my own
41. **Have you ever received education or guidance about medication use?** Yes / No
42. **Do you believe that educating people about medication adherence is important?** Strongly agree / Agree / Neutral / Disagree / Strongly disagree
43. **What type of information about medications would you find most useful?** (Select all that apply) How medications work / Side effects and management / Importance of adherence / Tools and strategies for adherence / Other
